# Supplementary material for: Resolving the full spectrum of human genome variation using Linked-Reads
Source: Genome Res. 2019 Apr;29(4):635–45. doi: 10.1101/gr.234443.118 (PMC6442396; doi:10.1101/gr.234443.118)
Supplement: Supplemental Material [file supp_gr.234443.118_Supplemental_longranger-2.2.zip › longranger-2.2.tar.bz2!/lr2.2-public/lib/stan/cmdstan/stan/doc/api/html/structstan_1_1gm_1_1validate__transformed__params__visgen.html]

Stan: stan::gm::validate\_transformed\_params\_visgen Struct Reference


|  |  |
| --- | --- |
|  | Stan  2.5.0  probability, sampling & optimization |


- Main Page
- Related Pages
- Namespaces
- Classes
- Files

- Class List
- Class Index
- Class Hierarchy
- Class Members

All Classes Namespaces Files Functions Variables Typedefs Enumerations Enumerator Friends Macros Pages

- stan
- gm
- validate\_transformed\_params\_visgen

Public Member Functions |
Public Attributes |
List of all members

stan::gm::validate\_transformed\_params\_visgen Struct Reference

`#include <generator.hpp>`

Inheritance diagram for stan::gm::validate\_transformed\_params\_visgen:

|  |  |
| --- | --- |
| Public Member Functions | |
|  | validate\_transformed\_params\_visgen (int indents, std::ostream &o) |
|  | |
| void | operator() (nil const &) const |
|  | |
| void | operator() (int\_var\_decl const &x) const |
|  | |
| void | operator() (double\_var\_decl const &x) const |
|  | |
| void | operator() (vector\_var\_decl const &x) const |
|  | |
| void | operator() (unit\_vector\_var\_decl const &x) const |
|  | |
| void | operator() (simplex\_var\_decl const &x) const |
|  | |
| void | operator() (ordered\_var\_decl const &x) const |
|  | |
| void | operator() (positive\_ordered\_var\_decl const &x) const |
|  | |
| void | operator() (row\_vector\_var\_decl const &x) const |
|  | |
| void | operator() (matrix\_var\_decl const &x) const |
|  | |
| void | operator() (cholesky\_factor\_var\_decl const &x) const |
|  | |
| void | operator() (cholesky\_corr\_var\_decl const &x) const |
|  | |
| void | operator() (cov\_matrix\_var\_decl const &x) const |
|  | |
| void | operator() (corr\_matrix\_var\_decl const &x) const |
|  | |
| void | validate\_array (const std::string &name, const std::vector< expression > &dims, size\_t matrix\_dims) const |
|  | |
| Public Member Functions inherited from stan::gm::visgen | |
|  | visgen (std::ostream &o) |
|  | |

|  |  |
| --- | --- |
| Public Attributes | |
| int | indents\_ |
|  | |
| Public Attributes inherited from stan::gm::visgen | |
| std::ostream & | o\_ |
|  | |

|  |  |
| --- | --- |
| Additional Inherited Members | |
| Public Types inherited from stan::gm::visgen | |
| typedef void | result\_type |
|  | |

## Detailed Description

Definition at line 1381 of file generator.hpp.

## Constructor & Destructor Documentation

|  |  |  |  |
| --- | --- | --- | --- |
| stan::gm::validate\_transformed\_params\_visgen::validate\_transformed\_params\_visgen | ( | int | *indents*, |
|  |  | std::ostream & | *o* |
|  | ) |  |  |

inline

Definition at line 1383 of file generator.hpp.

## Member Function Documentation

|  |  |  |  |  |  |
| --- | --- | --- | --- | --- | --- |
| void stan::gm::validate\_transformed\_params\_visgen::operator() | ( | nil const & |  | ) | const |

inline

Definition at line 1388 of file generator.hpp.

|  |  |  |  |  |  |
| --- | --- | --- | --- | --- | --- |
| void stan::gm::validate\_transformed\_params\_visgen::operator() | ( | int\_var\_decl const & | *x* | ) | const |

inline

Definition at line 1389 of file generator.hpp.

|  |  |  |  |  |  |
| --- | --- | --- | --- | --- | --- |
| void stan::gm::validate\_transformed\_params\_visgen::operator() | ( | double\_var\_decl const & | *x* | ) | const |

inline

Definition at line 1393 of file generator.hpp.

|  |  |  |  |  |  |
| --- | --- | --- | --- | --- | --- |
| void stan::gm::validate\_transformed\_params\_visgen::operator() | ( | vector\_var\_decl const & | *x* | ) | const |

inline

Definition at line 1397 of file generator.hpp.

|  |  |  |  |  |  |
| --- | --- | --- | --- | --- | --- |
| void stan::gm::validate\_transformed\_params\_visgen::operator() | ( | unit\_vector\_var\_decl const & | *x* | ) | const |

inline

Definition at line 1402 of file generator.hpp.

|  |  |  |  |  |  |
| --- | --- | --- | --- | --- | --- |
| void stan::gm::validate\_transformed\_params\_visgen::operator() | ( | simplex\_var\_decl const & | *x* | ) | const |

inline

Definition at line 1407 of file generator.hpp.

|  |  |  |  |  |  |
| --- | --- | --- | --- | --- | --- |
| void stan::gm::validate\_transformed\_params\_visgen::operator() | ( | ordered\_var\_decl const & | *x* | ) | const |

inline

Definition at line 1412 of file generator.hpp.

|  |  |  |  |  |  |
| --- | --- | --- | --- | --- | --- |
| void stan::gm::validate\_transformed\_params\_visgen::operator() | ( | positive\_ordered\_var\_decl const & | *x* | ) | const |

inline

Definition at line 1417 of file generator.hpp.

|  |  |  |  |  |  |
| --- | --- | --- | --- | --- | --- |
| void stan::gm::validate\_transformed\_params\_visgen::operator() | ( | row\_vector\_var\_decl const & | *x* | ) | const |

inline

Definition at line 1422 of file generator.hpp.

|  |  |  |  |  |  |
| --- | --- | --- | --- | --- | --- |
| void stan::gm::validate\_transformed\_params\_visgen::operator() | ( | matrix\_var\_decl const & | *x* | ) | const |

inline

Definition at line 1427 of file generator.hpp.

|  |  |  |  |  |  |
| --- | --- | --- | --- | --- | --- |
| void stan::gm::validate\_transformed\_params\_visgen::operator() | ( | cholesky\_factor\_var\_decl const & | *x* | ) | const |

inline

Definition at line 1433 of file generator.hpp.

|  |  |  |  |  |  |
| --- | --- | --- | --- | --- | --- |
| void stan::gm::validate\_transformed\_params\_visgen::operator() | ( | cholesky\_corr\_var\_decl const & | *x* | ) | const |

inline

Definition at line 1439 of file generator.hpp.

|  |  |  |  |  |  |
| --- | --- | --- | --- | --- | --- |
| void stan::gm::validate\_transformed\_params\_visgen::operator() | ( | cov\_matrix\_var\_decl const & | *x* | ) | const |

inline

Definition at line 1445 of file generator.hpp.

|  |  |  |  |  |  |
| --- | --- | --- | --- | --- | --- |
| void stan::gm::validate\_transformed\_params\_visgen::operator() | ( | corr\_matrix\_var\_decl const & | *x* | ) | const |

inline

Definition at line 1451 of file generator.hpp.

|  |  |  |  |
| --- | --- | --- | --- |
| void stan::gm::validate\_transformed\_params\_visgen::validate\_array | ( | const std::string & | *name*, |
|  |  | const std::vector< expression > & | *dims*, |
|  |  | size\_t | *matrix\_dims* |
|  | ) |  | const |

inline

Definition at line 1457 of file generator.hpp.

## Member Data Documentation

int stan::gm::validate\_transformed\_params\_visgen::indents\_

Definition at line 1382 of file generator.hpp.

---

The documentation for this struct was generated from the following file:

- src/stan/gm/generator.hpp


---

[ Stan Home Page ]

*© 2011–2014,
Stan Development Team.*
